# Paving the Way for Climate Neutral and Resilient Historic Districts

**DOI:** 10.12688/openreseurope.15392.1

**Published:** 2023-03-04

**Authors:** Aitziber Egusquiza, Daniel Lückerath, Saioa Zorita, Sophia Silverton, Gemma Garcia, Emilio Servera, Alessandra Bonazza, Igone Garcia, Antonis Kalis

**Affiliations:** 1 TECNALIA, Basque Research and Technology Alliance (BRTA), Derio, Spain; 2 Fraunhofer Institute for Intelligent Analysis and Information Systems IAIS, Sankt Augustin, 53757, Germany; 3 ICLEI European Secretariat, Freiburg, 79098, Germany; 4 Fundación de la Comunidad Valenciana para la Promoción Estratégica, el Desarrollo y la Innovación Urbana, Valencia, 46024, Spain; 5 Institute of Atmospheric Sciences and Climate, National Research Council, Bologna, Italy; 6 Institute of Communication & Computer Systems ICCS, Athens, Greece

**Keywords:** Resilience, heritage management, climate change adaptation, disaster risk management, historic district challenges, governance, knowledge co-production

## Abstract

Climate change is a major global threat to our society’s urban areas, with the majority of Europe's population living in cities and their cultural heritage. Historic districts of significant cultural value and the communities connected to these places have an important role to play in fostering location-based identity and economy, social cohesion, innovation, urban regeneration, and climate change adaptation. Thus, it is important to make historic districts climate resilient, by jointly considering climate change adaptation, disaster risk management, heritage management, and sustainable urban development. However, this is often a major challenge for local and regional administrators and relevant stakeholders.

This paper constitutes the first major result of the EU R&I Task Force for Climate Neutral and Resilient Historic Urban Districts. It provides an overview of the challenges faced by practitioners and researchers when jointly addressing the needs of resilient historic districts and provides an initial set of recommendations produced by the task force to address these challenges.

These challenges cover different issues around five topics (i) data availability, use and its management, (ii) the common responsibility fragmentation in policy and governance, (iii) the challenge on integrating local knowledge and traditions in resilience building, (iv) the difficulties around the co-ownership and co-production in governance and (v) the importance of mainstreaming heritage management in adaptation and resilience policies.

## Disclaimer

The views expressed in this article are those of the author(s). The sole responsibility for the content of this publication lies with the authors. It does not necessarily represent the opinion of the European Union. Neither the REA nor the European Commission are responsible for any use that may be made of the information contained therein. Publication in Open Research Europe does not imply endorsement of the European Commission.

## Introduction

Climate change is one of the biggest challenges facing our planet today. More frequent and intense natural hazards like droughts, heatwaves, floods, and storms are increasingly threatening species and habitats on a global and unprecedented scale. Cities are heavily affected by consequences of climate change, with most of Europe’s population living in cities and urban areas and projections for 2050 predicting even larger shares ^1^. At the same time, cities generate up to 80% of a country’s GDP ^2^ but also consume 75% of the natural resources and account for 60–80% of greenhouse gas emissions. That is, urbanisation and economic growth happening in cities are the biggest contributors to climate change. Adapting to urbanisation, climate change, digitalisation, and other social, economic and security trends is a challenging endeavour for cities and prone to potential conflicts of interest. It requires managing tasks like accommodating a growing and more diverse population, providing the required services, fostering social, environmental, and economic sustainability, and keeping the city liveable and attractive. But a liveable, sustainable and, above all, resilient city is not just a product of organised and well-functioning services: other crucial elements are the places that make up the city and the communities and their specific traditions that belong to those places. Historic districts of significant cultural value and the communities connected to these places have an important role to play in fostering location-based identity, social cohesion, creativity, innovation, urban regeneration, and climate change adaptation / mitigation. With the increased recognition of the threats from climate change these historic districts and their communities face, and the role they can play in driving climate action, everybody connected to historic districts faces both a major opportunity and a challenging responsibility ^3^.

To address these challenges and leverage the opportunities, the Horizon 2020 projects “Advancing Resilience of historic areas against Climate-related and other Hazards” ( ARCH), “A Decision Support System for Improved Resilience and Sustainable Reconstruction of historic areas” ( HYPERION), and “Sustainable Historic Environments hoListic reconstruction through Technological Enhancement and community based Resilience” ( SHELTER) have established the EU R&I Task Force for Climate Neutral and Resilient Historic Urban Districts.

The task force aims to bring together diverse groups of practitioners, researchers, and policy makers at the cross section of heritage management, climate change adaptation / mitigation, disaster risk management, and sustainable urban development. This with the objective to identify and discuss current developments in research and practice; bridge knowledge gaps between these fields; boost collaboration among the cross-sectoral actors involved; and ultimately make our cities more climate neutral and resilient.

In doing so, the task force aims to provide practical support to European authorities and decision makers for developing harmonised, evidence-based policies, strategies, and procedures. The technical core of the task force is made up of partners from European research projects and other interested organisations with relevance for resilient historic districts. In addition, practitioners and policy makers on European, national, and local level in fields related to resilience participate in the task force to discuss solutions offered by the technical partners and ensure their applicability.

This paper constitutes the first major result of the task force. It provides an overview of the challenges faced by practitioners and researchers when jointly addressing the needs of resilient historic districts and provides an initial set of recommendations produced by the task force to address these challenges. These recommendations are targeted at practitioners and policy makers on European, national, regional, and local levels involved in heritage management, climate change adaptation / mitigation, disaster risk management, and sustainable urban development, as well as researchers and funding bodies active in these fields.

To identify the challenges and produce the recommendations, the task force held three dedicated workshops over the course of 2021 and 2022:

- The *Task Force Kick-off Meeting* (23 ^rd^ June 2021) analysed the policy perspective for resilient historic districts, scientific gaps in achieving resilience for historic districts, and on-the-ground challenges for resilient historic districts.
- The s *econd workshop* (14 ^th^ – 15 ^th^ December 2021) examined cross-thematic problems, opportunities, and best practices from daily experience, as well as methods and tools to address problems and support opportunities.
- The *third workshop* (3 ^rd^ June 2022) refined the identified challenges and formulated initial recommendations to address these challenges.

Additionally, task force members participated in events of other initiatives, such as the Urban Agenda Partnership for Culture and Cultural Heritage, to discuss, refine, and align the findings from the workshops.

The remainder of this paper is structured as follows:

- We first provide the framework for the further discussions by introducing the concept of historic districts as social-ecological-technical systems, delimiting different definitions of resilience and how these definitions might be adapted to historic districts, and explain the connection between resilience climate change adaptation / mitigation and disaster risk management.
- Secondly, we locate the work of the task force in the policy landscape at the cross section of heritage management, climate change adaptation / mitigation, disaster risk management, and sustainable urban development.
- We then introduce the challenges for resilient historic districts identified by the task force before closing the paper with our recommendations to overcome these challenges and make the most out of the opportunities for resilience brought to the table by historic districts and their communities.

## Historic districts as social-ecological-technical systems, resilience concepts & their relationship with disaster risk management and climate change adaptation

Following UNESCO’s Recommendation on the Historic Urban Landscape ^4^, historic districts cannot simply be understood as a collection of buildings and structures, but rather as an amalgam of social-cultural-economic-governance systems – the social-economic domain – interacting with climate-biophysical-ecological and technological-engineered-infrastructural systems – the ecological-biophysical and technological-infrastructural domains ^5^. These domains have historic context and shape each other, not only in the past but also now and in the future. New developments in the different domains (be it urban development, climate change, or societal changes) reinforce and shape the roles and meanings they have for each other. Subsequently, historic districts cannot be seen as isolated systems, but as a holistic social-ecological-technical system (SETS) where heritage management, social and economic development, as well as disaster risk management and climate change adaptation / mitigation need to be integrated.

With climate change, natural and human-made hazards, development pressures, and other forces acting on the SETS, the resilience of these systems and their domains becomes of paramount importance. However, the term ‘resilience’ can mean many different things to many different actors depending on the context in which it is applied (see e.g. 6– 11). Broadly speaking, three different understandings of ‘resilience’ can be distinguished: engineering (or ‘narrow’) resilience, ecological / ecosystem and social resilience, and social-ecological resilience. While engineering resilience aims to withstand shocks and to return to a stable pre-disaster state as fast as possible e.g., ‘bouncing back’ ^11^, ecological / ecosystem and social resilience aims at adapting the system to better cope with the disaster (‘bouncing forward’, see e.g. 9). Social-ecological resilience in contrast, treats resilience as a process and acknowledges the need to account for uncertainty and include flexibility, learning, and the advancement of capacities and abilities of a system to withstand future shocks (see e.g. 9). This is also the view taken by the United Nations Office for Disaster Risk Reduction ( UNDRR) and the Intergovernmental Panel on Climate Change (IPCC), who in their 6th Assessment Report (AR6) ^12^ define resilience as:

*“[t]he capacity of social, economic and ecosystems to cope with a hazardous event or trend or disturbance, responding or reorganising in ways that maintain their essential function, identity and structure as well as biodiversity in case of ecosystems while also maintaining the capacity for adaptation, learning and transformation.”* (p.9)

However, while IPCC AR6 explicitly acknowledges the need of adaptation solutions to conform to the principle of justice and the value in diverse forms of knowledge, the propagated resilience definition still fails to explicitly link resilience and justice (as discussed in ARCH State-of-the-Art report no. 5 for AR5) ^13^, obscuring the fact that impacts are experienced by communities. Therefore, a definition of resilience for historic districts as SETS needs to embrace the concept of social justice and acknowledge that communities can be heterogeneous, exhibiting diverse needs, capacities, and levels of power.

Lastly, any resilience concept for historic districts needs to consider the specific characteristics of these SETS as well as the need to balance socially just response and adaptation with the need to maintain the historic district’s identity, integrity, and authenticity.

**ARCH, Resilience of a historic area**

*“The sustained ability of a historic area as a social-ecological system (including its social, cultural, political, economic, natural, and environmental dimensions) to cope with hazardous events by responding and adapting in socially just ways that maintain the historic area’s functions and heritage significance (including identity, integrity, and authenticity).”*

The complexity of resilience as a trans-disciplinary bridge between the fields of disaster risk management, climate change adaptation / mitigation and sustainable development (see also Morchain and Robrecht in 14), means that there has not emerged a consolidated definition yet, although these fields grow ever closer together – a topic the task force might tackle in future. However, ARCH and SHELTER have both suggested resilience definition more targeted towards historic districts as SETS:

**SHELTER, Resilience of a historic area**

*“Resilience of historic area refers to the ability of an historic urban or territorial system-and all its social, cultural, economic, environmental dimensions across temporal and spatial scales to maintain or rapidly return to desired functions in the face of a disturbance, to adapt to change, and use it for a systemic transformation to still retain essentially the same function, structure and feedbacks, and therefore the capacity to adapt in order to maintain the same identity”*

Addressing disaster risk reduces vulnerability, as do sustainable measures to deliver climate change adaptation (and mitigation, at least in the long term). These efforts enhance the resilience of SETS, including historic districts, and contribute to the sustainability of the system and to the long-term prevalence of culture, communities, economies, cities, and biodiversity, if they are shaped with sustainability criteria in mind ^14^. Resilient historic districts therefore require practitioners and decision makers to address both the long-term, slow onset future risks posed by climate change as well as the short-term sudden onset existing risks posed by disasters, whose intensity and frequency have already been increased by climate change. And in both cases, these risks must be addressed by reducing vulnerabilities and pursuing sustainable urban development as well as poverty reduction using ecosystem-based, engineered, social, economic, and institutional solutions that acknowledge how *“[c]ultural factors shape the [e]nabling conditions for adaptation and mitigation, including whether and how people respond to appeals for action.”* ^3^ In the context of historic districts, this needs to be understood to not just cover culture and arts but also sites of cultural heritage significance for the local community that play an important role in fostering place-based identity and social cohesion. Therefore, to make historic districts resilient, climate change adaptation / mitigation, disaster risk management, heritage management, and sustainable urban development need to be considered jointly.

**HYPERION, Resilience of a historic area**

*“Resilience of historic area refers to the measurable capability of the historic area to bounce back and recover from a disruptive natural hazard event. This capability is quantified based on metrics of physical damage, direct & indirect monetary losses, especially focusing on the Gross Value Added (GVA, which characterizes the economic output of the community) and evaluating it over the dimension of time.”*

## Policy landscape for resilience and historic districts

From the Sustainable Development Goals ^15^, the Paris Agreement ^16^ to the New Urban Agenda ^17^, resilience building in urban environments is a cross-cutting priority embedded in several international initiatives. However, most of these initiatives make no specific reference to historic districts or areas **.** Some efforts are made within the United Nations organisation through its Office for Disaster Risk Reduction, which promotes resilience building processes at multiple scales, including work at local level that also targets specific issues like cultural heritage. Current international efforts with city governments in relation to local disaster risk reduction and resilience are being developed through the Making Cities Resilient 2030 (MCR2030) multi-stakeholder initiative ^18^, running until 2030. MCR2030 aims at improving city resilience through easy access to tools and knowledge, some of which are applicable to historic districts or target cultural heritage.

At the European level, the European Union (EU) Civil Protection Mechanism was established by the European Commission in 2001 ^19^, involving not only EU countries but also additional participating states. Any country, in Europe and beyond, can request assistance through the Mechanism when a natural or human-made disaster exceeds its response capabilities. The EU Civil Protection Mechanism was upgraded in 2019 by the European Commission ^20^, when the rescEU additional capacities were established to provide faster and wider response to disasters and emerging risks. The important role of local authorities in disaster risk management is explicitly acknowledged in the Mechanism creation, as well as in its upgrade. The European Adaptation Strategy to Climate Change, approved in 2013, also recognised the need to translate its overall objective (to contribute to a Europe more resilient to climate change and variability) to the local level. After an evaluation in 2018, a new EU Adaptation Strategy was announced in 2019 by the European Commission in the European Green Deal. The strategy was adopted in 2021 ^21^, with the overall aim of adapting the European Union to the impacts of climate change by 2050, and the specific objectives of achieving such adaptation in smarter, faster, and more systemic ways, as well as increasing support for international climate resilience. Local adaptation action is one of the cross-cutting priorities identified within the systemic approach of the EU Adaptation Strategy. To achieve it, the need for increased EU support is recognised, i.e., via the strengthening of the EU and the Global Covenant of Mayors, or through the establishment of a policy support facility under the EU Covenant of Mayors.

Besides the EU Adaptation Strategy, other recent urban policies of the EU also highlight the need for more resilient and sustainable urban districts:

- The 2030 European Territorial Agenda ^22^ is a strategic policy document for spatial planning in Europe, its regions and communities. It provides a framework for action for territorial cohesion and calls on policy makers at all levels of governance to contribute to an inclusive and sustainable future for all places and to help achieve the Sustainable Development Goals in Europe.
- The New Leipzig Charter 2020 ^23^ provides a key policy framework document for sustainable urban development in Europe. The Charter emphasises that cities must establish integrated and sustainable urban development strategies and guarantee their implementation for the city, from its functional areas to its neighbourhoods.
- Declaration of Toledo 2020 on Urban Development focuses on how to face the present and future urban challenges of European cities and on how to apply the Europe 2020 strategy by achieving smarter, more sustainable and socially inclusive urban development. This new declaration strongly supports social innovation and its dissemination in the territory together with resilient economic systems.
- The EU Taxonomy for Sustainable Activities ^24^ is an EU-wide classification system for sustainable activities to scale up sustainable investment and to implement the European Green Deal.

The duration and magnitude of the COVID-19 crisis has reinforced the need to embed resilience into EU policy making. Temporary instruments such as the Recovery and Resilience Facility ^25^ have been established as part of the NextGenerationEU recovery plan, and resilience has begun to be monitored nationally through specific dashboards ^26^ that consider a broad set of indicators structured around four dimensions: socio-economic, green, digital and geopolitical.

While the need to consider resilience, climate change adaptation / mitigation, and disaster risk management as well as the role the local level needs to play in these fields, has clearly been recognised in international, European, and national policies and strategies, there is still a need for better addressing the specificities and potentialities of historic districts and cultural heritage.

## Challenges for climate neutral and resilient historic districts

During its one-year work across interdisciplinary workshops, participation in conferences and aligned initiatives, as well as via the experiences gathered throughout the work within its member projects, the task force identified five major challenge fields that need to be addressed for historic districts to become resilient.

### Challenge 1: Data and methods – access, harmonisation, usability

To make historic districts resilient, practitioners, researchers, and policy makers need reliable information that can inform decision making. This information currently needs to be collected from different sources, e.g., historical archives, earth observation data and products, census data, interviews with contemporary witnesses, climate model outputs, environmental monitoring, or other sources ^27, 28^. These sources might not be accessible to all relevant actors, might provide data in incompatible formats, data that spans different time intervals, or data with incompatible spatial resolutions. On top of this, required data might be incomplete or missing all together. This information from different sources and hugely diverse data sets of varying quality then needs to be harmonised, analysed, processed, verified, and understood to allow its use, e.g., in vulnerability, risk, and resilience assessments. Even if sufficient information is available, the knowledge derived from its analysis can often not be integrated in decision making processes, either because practitioners and decision makers lack the necessary background knowledge or support to make use of the knowledge, or the results of processing the gathered information are presented and communicated in ways not digestible and usable for decision makers. These challenges are made even harder by the inherent complexity of the trans-disciplinary concept of resilience (see section on SETS and resilience), which requires even more information from a wider selection of sources and targets a larger number of researchers, practitioners, and policy makers.

It is therefore not surprising that there is still a lack of standardised data formats and data gathering processes (which data is collected, at which time, and in which spatial resolution), although initiatives like the Infrastructure for Spatial Information in the European Community ( INSPIRE Directive) and the common European Data Space for Cultural Heritage are a step in the right direction. However, not only are standardised data sets missing, but the methods that make use of this data are also not harmonised sufficiently across different fields of expertise and differ depending on their aims and scope - a quantitative risk assessment at building-level might require different methods and data than an indicator-based risk assessment with lower-resolution at district-level. This lack of harmonisation makes it complicated to consistently combine methods across different scales – a necessity if a complete picture of the resilience of a historic district should be established ^29^ – and limits trans-disciplinary collaboration, as well as benchmarking and monitoring of resilience.

For historic districts, these issues made even more complex, because it is necessary to also integrate heritage values (socio-economic, intangible, or otherwise) with the fundamental data and analyse potential losses to these values in vulnerability, risk, and resilience assessments, which is a complex and often normative process.

As a result, researchers and practitioners need to make use of data, models and, tools with limited usability and reliability, need to either spend considerable effort to acquire large amounts of data for detailed assessments or employ less data demanding assessments that might not cover all necessary aspects, might need to conduct multiple assessments on different scales, and might need to translate results for different target audiences to provide actionable knowledge for decision making.

### Challenge 2: Fragmentation of responsibilities in policy and governance

Recent societies compartmentalise knowledge in the quest for expertise, resulting in siloed working approaches and a lack of common understanding of concepts, which does not help to build common strategies that could jointly address heritage management, disaster risk management, climate change adaptation / mitigation, and sustainable urban development. In other words, it impedes the cross-fertilization of solutions to create a holistic resilience strategy that can address the challenges associated with climate change and in the worst case can lead to detrimental overlapping of competences among decision makers on European, national, regional, and local level. Furthermore, apart from the knowledge fragmentation there is also a fragmentation of policy, which is often related to sectorial silos. However, although there is nowadays an effort to better account, coordinate, and integrate policies among different fields of knowledge, transversality is far from being a reality. For example, the integration of heritage management, disaster risk management, and climate change adaptation in mainstream policy is still incipient and rare are the examples in the EU landscape (i.e., National Plans of Adaptation to Climate Change in Italy and France ^30^). This fragmentation in policy has been observed at local, regional, and even national level. The different scale of the heritage management, disaster risk management and climate change adaptation policies results in additional challenges to define operative actions and specific protocols at local level.

### Challenge 3: Integrating local knowledge and traditions

Local knowledge and tradition are widely seen as important for resilience building in historic districts, influencing social behaviour, awareness, social capital, as well as supporting climate action and strengthening the local economy, among others. This includes not only the **use of traditional techniques**, e.g., in monument preservation, building construction, or sustainable agriculture and landscape protection, but also the **acknowledgement of the role local traditions**, like festivities or markets, as well as indigenous communities can play both in pre- and post-disaster contexts (‘Build Back Better’ phase). While there is some debate over the contemporary scientific validity of some traditional local knowledge, it is certain that the ‘intangible’ knowledge of a place’s past and current narratives is essential to societal resilience building. Using local knowledge from community stakeholders on climate change adaptation and mitigation is particularly valuable, and it builds inclusivity and ownership of people over their surroundings.

Although the value of local knowledge and traditions for resilience building are acknowledged, they are not yet consistently included by policy makers in climate change adaptation, disaster risk management, and sustainable urban development. Subsequently, the communities of historic districts are often not consistently engaged in resilience building actions which could hugely benefit from their participation, e.g., training activities for recovery of build materials, reconstruction activities using traditional building techniques, or traditional landscape maintenance, cultivation, and use ^31^.

Another open question remains, as to how to include local knowledge and traditions, which often takes the form of narratives or storytelling, with quantitative approaches in disaster risk management, climate change adaptation, heritage management, and sustainable urban development. This issue is strongly linked with the question of how to better approach and engage local communities in knowledge co-production (e.g., for risk analyses), also considering requirements of and challenges for diverse social groups (e.g., limited accessibility to information, events, tools, etc. due to language barriers, limited comprehension of digital technologies, social constructs, disabilities, and more).

### Challenge 4: Co-ownership and co-production in governance

There are several challenges embedded in the governance of resilient historic districts. Involvement of diverse local communities and stakeholders in governance processes faces competition for attention between different initiatives, accessibility barriers due to complexity of approaches, lack of resources, suitable expertise, and a common language, as well as scepticism regarding the usefulness and availability of initial results, and scepticism about if and how input from local stakeholders will be used in decision making processes. Notably, it is an ongoing challenge to find individuals who will take specific responsibility over research results. This implies a governance gap between research and practice.

Despite these complexities, input from local communities and stakeholders should not be excluded from decision making processes that should be based on a user-driven approach and addressed to provide solutions to the territorial challenges. A good governance process considers local values, risk perceptions, and priorities around climate change impacts and responses, valuing long-term increases in resilience over short-term profit. Co-creation and awareness strategies are still not empowering people and communities enough to be a part of the solution. Perhaps more importantly, decision makers and authorities often are not prepared to accept increased empowerment of people and communities. In the governance framework lacking financing and investment possibilities (i.e., nature conservation, ecosystem restoration, water management, climate change adaptation, infrastructure maintenance) influence the opportunities for heritage resilience.

### Challenge 5: Mainstreaming heritage management and resilience

Heritage could be a powerful contributor to resilience building, not only as an asset to protect, but as a dynamic part of the solution. Heritage can, for example, generate awareness of tangible direct and indirect impacts of climate change, particularly when monuments and archaeological sites are irreversibly damaged by extreme hazards, like flash flood, storm surges, and fire, or submerged by sea level rise. As large parts of society are often passionate about heritage (especially tangible assets) and willing to give time or money to help protect it, heritage issues can galvanise communities into action more than many other matters.

Heritage sites can also offer important insights for climate change adaptation, e.g., by providing examples for more sustainable adaptation measures based on local materials and skills or by adapting traditional building techniques from one climatic zone to adapt buildings in another geographic regions that might in future exhibit a similar climate. Unfortunately, these potentials offered by the heritage sector are **often not acknowledged or prioritised** by those involved in climate change adaptation, disaster risk management, and sustainable urban development.

On the other hand, heritage can only support transformational changes if theoretical ideas quickly become actionable strategies. Yet, the heritage sector does not have a reputation for flexibility and openness to change. Contributing factors for this issue can also be land use, landscape configuration, geomorphology, and urban morphology, which can limit the capacity for action and flexibility for defining adaptive solutions. Particular difficulties can be observed when trade-offs exist between adaptation and preservation requirements.

## The way forward: recommendations for climate neutral and resilient historic districts

### Challenge 1: Data and methods – access, harmonisation, usability

To address the challenges associated with access, harmonization, and usability of data and methods, three fields of actions should be pursued:

(1) Improve access to reliable data with harmonised formats, gathered in a consistent way across multiple scales. To increase the availability and consistency of data at different spatial scales, a multi-level initiative to harmonize data formats and acquisition processes on European, national, regional, and local levels should be initiated. This initiative should start from the INSPIRE Directive – which the European Commission plans to revise soon - and the common European Data Space for Cultural Heritage, which should start to be deployed over the next two years and should make high-value datasets on cultural content available. This initiative should also address issues like integrating data from different sources (e.g., local-level sensor data on air pollution with European level data from the European Environment Agency). While this initiative should be initiated in a top-down fashion (on European and national level), it is paramount to include the operational level and local population at appropriate stages of the definition, design, and data acquisition process in a bottom-up fashion. This could take the form of crowd sourced data, participatory sensing, and civic science, which would have the co-benefit of increasing the involvement and empowerment of the local population, helping to also address challenges 3 and 4. In addition, this initiative needs to also include different disciplines, from social sciences and history to urban development, climate science, computer science, engineering, and material science to increase data quality.
Based on this initiative, the urban data platforms that are often already available on municipal level need to be extended towards public resilience observatories, making data for climate change adaptation / mitigation, disaster risk management, heritage management, and sustainable urban development available and (dis)aggregating the data available on European, national, and regional levels for use on the local level. Such data would, for example, enable the creation of multi-layered digital twin models / tools for historic districts, including structural details, infrastructure networks (e.g., transport, power, water networks) together with economic activity models.
(2) Advance the harmonization of methods, the integration of heritage values and subsequently enhance the usability and reliability of information. To advance the harmonization of methods and the integration of heritage values, more research is needed on multi-level assessment approaches that combine quantitative and qualitative data as well as assessments on heritage values, losses impacts and deterioration processes. The Impact Chain approach for climate risk and vulnerabilities assessments ^32^ could be a good starting point. Impact Chains, which are based on the SETS framework, are usually developed in a multi-stakeholder process, and can model complex, cascading cause-effect relationships between climate impacts and risks, provide an easy to use and understand communication tool, and can be used as the backbone of an operational risk assessment. They can help address the need for easier to understand risk assessment methods for heritage practitioners, as well as combining quantitative and qualitative approaches – both using indicator-based as well as more sophisticated quantitative approaches. Another starting point could be the “Risk Mapping Tool for Cultural Heritage Protection” developed in the framework of the Interreg Central Europe STRENCH project, which provides a methodology for hazard-oriented vulnerability ranking for diverse categories of cultural heritage. Both approaches offer a way to further harmonize methods for vulnerability, risk, and resilience assessment across the fields of heritage management, climate change adaptation, and disaster risk management.
Regardless of the entry point for harmonization, additional research is also needed on how to co-identify and co-evaluate heritage values in multiple dimensions (social, cultural, artistic, economic, etc.), and how to integrate these heritage values - and the potential loss of these - into approaches for vulnerability, risk, and resilience assessment.
(3) Provide more and better training, education, and capacity building opportunities on how to make use of data and results, in decision making and how to provide information in formats suitable for decision makers. For the practitioner side, it is necessary to provide specific training opportunities as a permanent option. This includes the use, implementation, and combination of different assessment methods, research results in general, as well as the use of available public data, e.g., from Copernicus ^27^, the future European Data Space for Cultural Heritage, as well as potential national, regional, or local resilience observatories. In conjunction with increased training opportunities for practitioners, researchers need to be able to provide their findings in a language and format appropriate for the relevant audience. Funding bodies as well as academic / research institutions should incentivise societal impact of research even more. The Horizon Result Booster of the European Commission is a step in this direction, but currently usually focused on communication, dissemination, and exploitation of research outputs. It would be beneficial to extend the Horizon Result Booster with an additional service that specifically supports highly interdisciplinary research projects in how to translate complex research outputs, i.e., supporting experts from different fields in translating their knowledge into digestible and usable formats for experts from other fields.

### Challenge 2: Fragmentation of responsibilities in policy and governance

To reverse the adverse effects of knowledge and policy fragmentation three main strategies have been suggested to raise awareness:

(1) **Harmonisation and standardisation of terminology and practices**. More effort must be made to develop a common vocabulary that shares concepts concerning climate change, the environmental field, cultural heritage, and governance processes, since *“disciplines are themselves societies, each with its own unique cultural content and linguistic code of signs, symbols, and syntax”* ^33^. This approach could be tackled, among other activities, by formal and informal standardisation activities as concepts, terminology, and management frameworks could be consolidated through guidelines and standards.
(2) **Co-ownership of the resilience goals and management strategies.** All parties involved in heritage management, climate change adaptation, disaster risk management, and sustainable urban development, among others, must be conscious of, and collectively work towards a common resilience goal, however loosely defined that goal might be. A starting point could be to tackle one common strategy such as an adaptation strategy which could help in creating awareness at the wider political level. A legislative framework could be established to further promote cross-sectoral communication and cooperation on a regular basis among all interested parties. And one step forward for silo breakdown can be the development of resilience teams with shared responsibility and budget management to carry out cross-sectoral projects to achieve the previously identified resilience goal.
(3) **Raise awareness at policy level** of the importance to protect culture and cultural heritage and decrease its vulnerability towards natural and human-made disasters by putting forward dedicated measures and actions to be included in the existing national plans for adaptation to climate change and disaster risk reduction and management.

### Challenge 3: Integrating local knowledge and traditions

A two-pronged strategy should be taken to address better inclusion of diverse forms of local knowledge and traditions in resilience planning:

(1) Engagement techniques for participative methods, e.g., in risk assessment or adaptation planning, should be better tailored to the relevant community groups and their diverse members to better capture and include local knowledge. This can mean making use of social networking tools that can attract people to the topics of heritage management, climate change adaptation / mitigation, disaster risk management, and sustainable urban development and which can also encourage people to provide different types of input to relevant processes – from using participatory sensing for gathering quantitative data to ways for people to provide photos, videos, oral testimonies, and other more qualitative data. However, engagement techniques need to be specifically tailored to the members of the local communities, which can also mean that more traditional ways of engaging people need to be explored, e.g., interviews, surveys, workshops, and more. In addition, it can help to engage local communities within their existing structures (e.g., churches, associations, community groups).
(2) The inclusion of local knowledge requires **more research into mixed-method approaches** (see also recommendations for challenge 1), e.g., for risk analyses, to design better and more consistent methods for combining qualitative and quantitative data as well as fusing knowledge from multiple perspectives. Not only can this increase the validity of results from assessments by linking them to experiences “on the ground”, but it can also open potential new avenues for resilience planning and increase the acceptance of required measures.

An approach that can support both recommendations above, is the incorporation of narratives and storytelling both as a means for better engagement and a way to include diverse knowledge in assessments approaches, e.g., via gaming or other means. Use of narratives and storytelling in different forms allow to easily capture qualitative information from local community members, increase the engagement of local communities, and can also make it easier to communicate complex topics such as resilience. These approaches also can have the co-benefit of allowing to include the culture and art sector, which is exceptionally experienced in capturing and crafting stories (see also recommendations to challenge 5).

On top of the two-pronged approach above, policy and decision makers need to be better incentivised to include the use of traditional techniques in climate change adaptation, disaster risk management, and sustainable urban development. For example, this could take the form of specific requirements for planning processes (e.g., for climate change adaptation) to evaluate the use of local, traditional techniques as alternatives to other resilience building measures. Other approaches could be specific funding schemes for the inclusion of traditional, local knowledge, or requirements to involve local communities in planning and training activities for post-disaster recovery.

### Challenge 4: Co-ownership and co-production in governance

The governance challenge is closely connected to the local knowledge challenge. As such, the recommendations on better and more tailored engagement techniques are also valid here. As part of the contemporary technological environment that is now constantly present in our everyday activities and our culture, these community engagement tools should act as a vessel for pre-disaster, post-disaster, and during-disaster engagement, enhancing the idea that heritage is not a thing of the past, but more of a foundation for actively responding to unforeseen challenges of the future.

In addition to these recommendations - and regardless of engagement technique - experts in charge of resilience planning need to engage local communities as closely as possible. This goes beyond inviting citizens and local communities to ‘public consultations’ organised by the district or municipality within their own facilities, as these meetings will mostly attract the “usual suspects”. Instead, those in charge of resilience planning must make the effort to engage local communities on their terms, e.g., during community gatherings, local festivities, and other events where the diverse group of community members can participate jointly. This will require those in charge to be equipped with sufficient personnel and funding and the clear mission to engage local communities.

To further facilitate stronger involvement of local communities, the areas of heritage management, climate change adaptation / mitigation, disaster risk management, and sustainable urban development need to be re-designed to allow increased involvement of local communities and facilitate co-creation of processes and measures wherever possible. Thus, policy makers as well as the researchers and practitioners consulting them in strategy development need to shift the focus of policy and research-policy strategies from the often strong and narrow economic-technological aspect to a focus on the whole social-ecological-technical system. Subsequently, funding bodies need to require research projects to *“incorporate more heterogeneous actors to foster inter- and transdisciplinary knowledge co-creation. These actors may need to be different in age, gender, social and educational background in order to allow for different solution options and overcome paradigmatic “lock-in” in unsustainable value systems as well as the issue of bounded morality of systemic actors”* ^34^ (p. 9). More specifically, research projects need to be designed to be more inclusive and make stronger efforts to include representatives of those communities they are supposed to serve. This could also lower the barriers to take ownership of research results.

At the same time, project coordinators and partners in charge of knowledge co-production need to be mindful that some communities might not have the capacity to concern themselves with the issues at the heart of a research project, as their main issues might be more existential (e.g., in deprived areas the priorities of residents might be survival of their families rather than their neighbourhood heritage). However, in some of these cases heritage might also offer a solution to better engagement while simultaneously connecting past and future. An example for such an approach can be using urban agriculture to empower citizens to practice organic and regenerative horticulture using traditional crops/local varieties

While such participative processes take time, they are especially important when resilience measures target historic districts. Only if communities are included in all phases of the resilience planning process (before, during, and after disaster) will potential resilience enhancing measures - and potential changes to the historic district incurred by these measures - be accepted by the communities. This also includes the need to broadly co-identify what is protection worthy, which risk levels might be acceptable, and how to cope with the dynamic nature of development in municipal districts together with local communities.

However the increased need for more knowledge co-production with diverse community groups also comes with a price when it comes to research projects: With research programmes on European level covering broader topics and requiring more transdisciplinary consortia, including social science, climate science, engineering, computer science, material science, as well as representatives from civil society and industry, while simultaneously limiting the number of funded projects further compared to previous research programmes, successful research projects often have to promise more and increasingly complex results under limited budgetary capacities of individual partners. This can in turn limit how intense partners are able to interact, how agile the knowledge co-production process can be designed, and how far-reaching engagement processes outside of projects can be, subsequently influencing the quality of outputs. Here, funding bodies should make sure that the required highly transdisciplinary research projects that require large consortia and diverse community groups receive sufficient funding (for all partners), reducing the temptation for project consortia to over-promise due to high competitiveness and an unrealistic amount of expected project outcomes.

Beyond stronger community involvement and sufficient funding for research projects, the processes for heritage management, climate change adaptation, disaster risk management, and sustainable urban development need to be better integrated. Mayor *et al.*, 2021 ^35^ argue that integrated spatial and urban planning and adaptive management approaches have the potential for transformational changes, facilitating the deployment of measures for climate change adaptation and disaster risk management, including for instance nature-based solutions, as well as enabling the mobilisation of the resources that support their effective implementation. They base their argumentation on the following points:

- Integrated spatial and urban planning are transversal disciplines that address socio-economic and environmental issues in balance with sustainable development ^36, 37^.
- Planning departments and technical teams do have the knowledge and understanding of the territorial and urban reality, usually working at the interface between the environment, the social needs, and the market, thus they could also boost new ways of green investment ^35^.
- Formal and institutional planning do also have the potential to anchor planning guidelines, criteria, and standards for local climate adaptation ^37^.
- Local governments have a key role in the design of projects to help in the transformation of urban areas towards more sustainable solutions. Depending on the administrative structure and the distribution of powers and responsibilities, many local authorities may have resources and capacity for climate action, especially relevant from the perspective of adaptation, through local policies such as urban planning, drinking water supply, sanitation networks and wastewater treatment, the management of roads and public spaces, green public areas, environmental protection, or public health ^36^.

Acknowledging the different planning approaches and systems in place would allow us to (i) anticipate the potential barriers for the implementation of certain business, governance, and financial models, and (ii) identify the opportunities and specific mechanisms that would facilitate the articulation of those models ^35^.

This re-design of planning processes and community engagement needs to be flanked by more comprehensive communication and awareness raising campaigns specifically tailored to different community groups and their members. An example for such measures could be outreach activities at schools to inform about the work involved with disaster risk management.

### Challenge 5: Mainstreaming heritage management and resilience

To increase the role heritage can play in resilience planning, several recommendations can be made. First, those involved in climate change adaptation / mitigation, disaster risk management, and sustainable urban development - from policy and decision makers to researchers - need to make better use of the heritage sector, including culture and creative industries, in creating momentum for climate and disaster action. On the one hand, this can take the form of using the unique values of heritage - and the potential loss of these - as a communication tool for creating urgency, but also hope, in messaging, as these heritage assets have often withstood multiple disasters over their lifespan. On the other hand, the culture and creative industry is uniquely positioned to support the need for more storytelling and narratives for better community engagement and communication in resilience planning and assessment processes. This is especially powerful in cases of heavy disasters in the past, which often become common memory of a region and subsequently intangible heritage as well (e.g., a museum on the storm surge in 1962 in Hamburg, is in preparation to document that night with almost 350 deaths and its outcome after 60 years of an ongoing disaster risk management on high water events in Hamburg).

However, the inclusion of the heritage sector should not just stop at message crafting. Instead, the culture and heritage sectors should routinely be involved in climate resilience planning and actions at all levels to ensure related actions are in line with the – community-agreed – protection goals as well as local traditions. At the same time, the heritage sector might need to move away from its strict focus on preservation and - especially in the face of the accelerating climate crisis - make engagement with disaster risk management, climate change adaptation, and urban planning colleagues an integral part of its practices.

To facilitate a joint better understanding, it is necessary to provide training and knowledge exchange, both to heritage managers on topics like climate change adaptation and disaster risk management, but also to climate change adaptation and disaster risk management professionals on relevant heritage management topics. Such training would also foster better mutual understanding as well as harmonisation between approaches and terminology (see also recommendations for challenge 2).

Ultimately, the mutual engagement and training provision should result in the establishment of a joint resilience team or office at local level with an official mandate to coordinate the resilience planning process across all involved departments.

## Ethics and consent

Ethical approval and consent were not required.

## Data Availability

No data are associated with this article.
