# Supplementary material for: Paving the Way for Climate Neutral and Resilient Historic Districts
Source: Open Res Eur. 2023 Mar 4;3:42. [Version 1] doi: 10.12688/openreseurope.15392.1 (PMC10521047; doi:10.12688/openreseurope.15392.1)
Supplement: Peer review history

# Reviewer report: Reviewer response for version 1

**DOI:** 10.21956/openreseurope.16638.r34987

**Published:** 2023-09-18

**Authors:** Giulia Ulpiani

**Affiliations:** 1 European Commission, Joint Research Centre (JRC), Ispra, Italy

In this paper, the thorny question on how to make historic districts climate resilient is addressed by highlighting key challenges and proposing recommendations.

 In its current form, the manuscript comes with three major shortcomings that need to be addressed (especially point 1):

1. The title is “Paving the Way for Climate Neutral and Resilient Historic Districts”, however no mention to climate neutrality is made in the abstract and emissions reduction is not included in the five topics listed at the end of the abstract. This imbalance is reflected throughout the manuscript. As-is, the manuscript cannot claim to tackle climate mitigation, emissions reduction, and/or climate neutrality. The authors are required to change the title to reflect the real scope of their work.
2. The article fails at linking own results with results of existing literature, hence no real discussion is presented in any section of the manuscript.
3. Similarly, the article comes with no literature review (describing the policy context is not considered a literature review), and resultantly, it stays disconnected from the knowledge, results, and recommendations extracted elsewhere in the scientific community.

Other (minor) comments are presented below on a section-by-section basis:

 Introduction

- …“but also consume 75% of the natural resources and account for 60–80% of greenhouse gas emissions.” No reference provided for this.
- …“boost collaboration among the cross-sectoral actors involved; and ultimately make our cities more climate neutral and resilient.” A reference could be made here to the Horizon Europe 100 Climate-Neutral and Smart Cities Missions as well as to the Mission on Adaptation to Climate Change, as these are two flagship programs championing both emissions reduction and climate resilience in cities. For instance, a recent publication on the Cities Mission revealed that, among highly ambitious cities in climate action aiming for climate neutrality by 2030, less than 50% focus on historical buildings. This could support the importance of conducting the proposed research, but can also be mentioned in Section “Policy landscape for resilience and historic districts” ^1^.
- The method (e.g. the workshops and the underlying analyses that formed part of the three projects) could and should be dealt with in a dedicated section.
- No literature review (of existing studies dealing with same or similar topic) is included in the introduction hence, there is no scene set for the activities of the Task Force. A concise literature review should be included.

Section: “Historic districts as social-ecological-technical systems, resilience concepts & their relationship with disaster risk management and climate change adaptation”

- Consider shortening the section heading.
- Page 4, the IPCC quotation seems off. It should be “The capacity of social, economic, and environmental systems to cope with a hazardous…”
- This section is somewhat repetitive in content with large space devoted to offering different definitions and multiple concluding remarks on the (same) need to blend climate change adaptation / mitigation, disaster risk management, and sustainable urban development. It should host a deeper reflection on what makes historic districts such a special case when it comes to climate resilience (and other co-targets).

Section: Challenges for climate neutral and resilient historic districts

- “For historic districts, these issues made even more complex, because it is …”, verb seems missing.
- “In the governance framework lacking financing and investment possibilities (i.e., nature conservation, ecosystem restoration, water management, climate change adaptation, infrastructure maintenance) influence the opportunities for heritage resilience.” Please check this sentence.

Where applicable, are recommendations and next steps explained clearly for others to follow? (Please consider whether others in the research community would be able to implement guidelines or recommendations and/or constructively engage in the debate)

Yes

Does the article adequately reference differing views and opinions?

No

Are all factual statements correct, and are statements and arguments made adequately supported by citations?

No

Is the rationale for the Open Letter provided in sufficient detail? (Please consider whether existing challenges in the field are outlined clearly and whether the purpose of the letter is explained)

Partly

Is the Open Letter written in accessible language? (Please consider whether all subject-specific terms, concepts and abbreviations are explained)

Yes

Reviewer Expertise:

Climate neutrality and adaptation in cities, urban climate, energy efficiency and conservation in cities, building physics

I confirm that I have read this submission and believe that I have an appropriate level of expertise to confirm that it is of an acceptable scientific standard, however I have significant reservations, as outlined above.

## References

[1] : Towards (net) zero emissions in the stationary energy sector: A city perspective. *Sustainable Cities and Society* .2023;97: 10.1016/j.scs.2023.104750 10.1016/j.scs.2023.104750

# Reviewer report: Reviewer response for version 1

**DOI:** 10.21956/openreseurope.16638.r32144

**Published:** 2023-09-05

**Authors:** Nicole Heller

**Affiliations:** 1 Anthropocene Studies, Carnegie Museum of Natural History, Pittsburgh, Pennsylvania, USA

Egusquiza et al. identify the overlapping agendas and actors involved in adapting urban historic districts to climate change. They present five challenges in advancing integrated resilience planning and action, as well as recommendations for addressing these challenges. Lessons are targeted to policy makers, researchers and practitioners. Challenges and recommendations were identified through workshops that took place over one year, developed as part of an EU R&I Task Force for Climate Neutral and Resilient Historic Urban Districts, which brought together “diverse groups of practitioners, researchers, and policy makers at the cross section of heritage management, climate change adaptation / mitigation, disaster risk management, and sustainable urban development.” The inclusion of diverse people working across research, practice and policy is an excellent step to advance more holistic planning. The findings will be most relevant for European researchers, planning and policymakers, ideally stimulating more cooperation among government, academia and the cultural heritage sector.

 While the paper is European focused and indexes many specific EU policies and toolsets, I think it is useful for those in other sectors and regions as these issues are generalizable across countries and planning sectors. As a researcher and climate adaptation practitioner working in the US, I recognize all 5 of the challenges identified. These are the big issues that need to be addressed in climate change adaptation: harmonizing data, coordinated regional planning, co-production, inclusion/justice, and mainstreaming. I have observed all 5 challenges in my research and practice working on adaption in the land, water and biodiversity conservation sector in the US. And indeed from that point of view, it is important to recognize that these problems have been acknowledged for a long time, and still remain quite difficult to overcome. So while I appreciate the authors points and agree, the challenge will be in making these recommendations actionable. From that point of view, I wish the authors would have done more to illuminate learning from the broader literature and technical work in other regions and sectors.

 Greater engagement with the learning from other regions/and sectors about how others have tried to address these challenges would help the European effort. As the authors discuss, this work is complicated, and especially so in the heritage sector. I would like to see some case studies and attention to how other regions or sectors are working to develop methods, tools and support to address these challenges and harmonize city planning. For example, in the case of harmonizing data, personal experience demonstrates that this harmonization is a time consuming problem to address at scale. Even once data is standardized and made available, practitioners and policy-makers often don’t use the data, or don’t know how to use the data. This need for translation services has been a widely recognized issue for decades, and one of the impetuses for co-production methods in climate adaptation. Consequently, I suspect there is more that could be reviewed about how to best do this from cases published in the literature and from how other agencies/groups (e.g. boundary organizations) have formed to meet this need.

 That being said, I agree entirely with the authors recommendations that the cultural heritage sector is especially well positioned to play a greater role in spurring integrated resilience planning, community engagement and empowerment. A key role for museums and other cultural institutions at this time is supporting civic life and serving as boundary organizations bridging technical and policy work with community input, memory and storytelling. 

 A second area of the paper that is well-framed, but also would benefit from more analysis relates to the integration of the social and ecological. Despite the presentation of historic districts as social-ecological-technical systems, this review focuses mostly on buildings and structures, with very little discussion of landscapes, parks, open-spaces, and ecological datasets and values. Yet the ecological/land side of the equation is not only critical for ecosystem-based adaptation, but also has co-benefits to plants, wildlife, biodiversity, and the well-being of people living in cities. In revision or follow-up work, I would like to see more analysis of the social *and *ecological dimensions of adapting cultural heritage districts, how these different domains can be better integrated, and specific opportunities to advance multiple ecological and social sustainability goals in adapting historic districts to global change. Some case studies and conceptual diagrams would probably help to illuminate the overlapping sectors and actors and opportunities for integration.

 I found the presentation of cities with regard to carbon emissions to be partial. In the introduction, cities are presented only as contributors to climate change. The authors should qualify their remarks about urban emissions to acknowledge the role urban areas play in supporting low-carbon lifestyles. Per capita emissions are lower in cities compared to national averages, and higher density cities have lower per capita emissions. The average per capita emissions of people in cities are lower than for those living in rural or suburban areas. The form of human dwelling matters, and this variation is a relevant component of climate adaptation/mitigation. The UN 2011 report cited in the paper discusses how urban areas generate emissions, but also harbor the majority of populations in small areas. In revisions, I recommend the authors revise their introduction to cities, and their overall analysis, to weave a more nuanced discussion of carbon emissions and urbanism, and also to explore how carbon mitigation and the resilience of historic districts. How might historic districts attract urban living more generally and encourage the low carbon lifestyles of urban residents? In the mainstreaming heritage section, the authors discuss how cultural institutions are particularly well-suited to advance education efforts about climate adaptation in relation to extreme events. I would add they are also useful to demonstrate and promote mitigation with the general public. See for example, Julie’s Bicycle out of the UK which promotes sustainability toolsets for the arts and culture sector. This is an additional aspect of why the cultural heritage sector is an essential part of integrated, holistic adaptation and mitigation planning and community engagement.

 Finally, I found the attention to resilience and social justice exciting to see, but also ripe for further development. The authors spend some paragraphs discussing resilience definitions and how they can be strengthened. I appreciated the authors attention to integrating social justice into resilience definitions, and the recognition of the heterogeneity and power differences that exist within and among communities. I welcome the call for more mixed-method approaches to integrate local knowledge. These are very important points, which raise challenging questions, such as: Can social justice be addressed while also retaining the essential identity, integrity, and authenticity of a historic district? Are “authenticity” and “preservation” too inflexible to allow adaptation of historic districts? Is the heritage sector too conservative to adapt? I have analyzed a related paradox in the management of natural areas and national parks, where managers try to restore historic ecological conditions, but this can fail to provide flexibility for ecosystems to adapt and change over time (Heller and Hobbs 2014) ^1^. In revisions, I would like to see more connections made between calls for social justice and the authors points on integrating local knowledge and traditions and co-production. Indeed if these are already challenges, and as they write “decision makers and authorities often are not prepared to accept increased empowerment of people and communities.” This will only get more difficult as more heterogenous and marginalized groups are engaged. Integration of social justice into resilience goals will ideally reveal synergies to improve sustainability and social justice, but it will also introduce difficult tradeoffs and barriers. Can the authors anticipate how much of a problem this might be with the specific case of historic districts in EU cities? How might cultural institutions help with these deliberations? Struggling through social and ecological frictions will be essential for building social-ecological resilience in practice, and practitioners and policy makers will need support to do this.  

 Small points:

 The authors reference a number of toolsets (ARCH, HYPERION, SHELTER). For readers not familiar with these efforts, it would be helpful to have some description of each of these. 

 This statement would benefit from a citation: “While there is some debate over the contemporary scientific validity of some traditional local knowledge”.

Where applicable, are recommendations and next steps explained clearly for others to follow? (Please consider whether others in the research community would be able to implement guidelines or recommendations and/or constructively engage in the debate)

Partly

Does the article adequately reference differing views and opinions?

Partly

Are all factual statements correct, and are statements and arguments made adequately supported by citations?

Partly

Is the rationale for the Open Letter provided in sufficient detail? (Please consider whether existing challenges in the field are outlined clearly and whether the purpose of the letter is explained)

Yes

Is the Open Letter written in accessible language? (Please consider whether all subject-specific terms, concepts and abbreviations are explained)

Yes

Reviewer Expertise:

Climate adaptation, social-ecological systems, museum studies

I confirm that I have read this submission and believe that I have an appropriate level of expertise to confirm that it is of an acceptable scientific standard, however I have significant reservations, as outlined above.

## References

[1] : Development of a natural practice to adapt conservation goals to global change. *Conserv Biol* .2014;28(3) : 10.1111/cobi.12269 696-704 10.1111/cobi.12269 24617971

# Reviewer report: Reviewer response for version 1

**DOI:** 10.21956/openreseurope.16638.r30963

**Published:** 2023-03-14

**Authors:** Mariella de Fino

**Affiliations:** 1 Department of Civil, Environmental, Land, Building Engineering and Chemistry (DICATECh), Polytechnic University of Bari, Bari, Italy

The article is a very well structured and presented overview of the challenges posed by climate change resilience in historic districts, on the basis of the interesting and inspiring results of the EU R&I Task Force for Climate Neutral and Resilient Historic Urban Districts. The key aspects that need to be addressed by practitioners and researchers for historic districts to become resilient are clearly identified and supported by sound scientific and institutional references. Moreover they are rigorously linked to general recommendations and actions that cover the multi-disciplinary and multi-scale dimension of the problem.

Where applicable, are recommendations and next steps explained clearly for others to follow? (Please consider whether others in the research community would be able to implement guidelines or recommendations and/or constructively engage in the debate)

Yes

Does the article adequately reference differing views and opinions?

Yes

Are all factual statements correct, and are statements and arguments made adequately supported by citations?

Yes

Is the rationale for the Open Letter provided in sufficient detail? (Please consider whether existing challenges in the field are outlined clearly and whether the purpose of the letter is explained)

Yes

Is the Open Letter written in accessible language? (Please consider whether all subject-specific terms, concepts and abbreviations are explained)

Yes

Reviewer Expertise:

Energy assessment and retrofitting of historic constructions; Building diagnostics and monitoring; Digital tools for heritage documentation, informative modelling and risk management.

I confirm that I have read this submission and believe that I have an appropriate level of expertise to confirm that it is of an acceptable scientific standard.
